# Supplementary figures and images for: Rab8, Rab11, and Rab35 coordinate lumen and cilia formation during zebrafish left-right organizer development
Source: PLoS Genet. 2023 May 15;19(5):e1010765. doi: 10.1371/journal.pgen.1010765 (PMC10212091; doi:10.1371/journal.pgen.1010765)

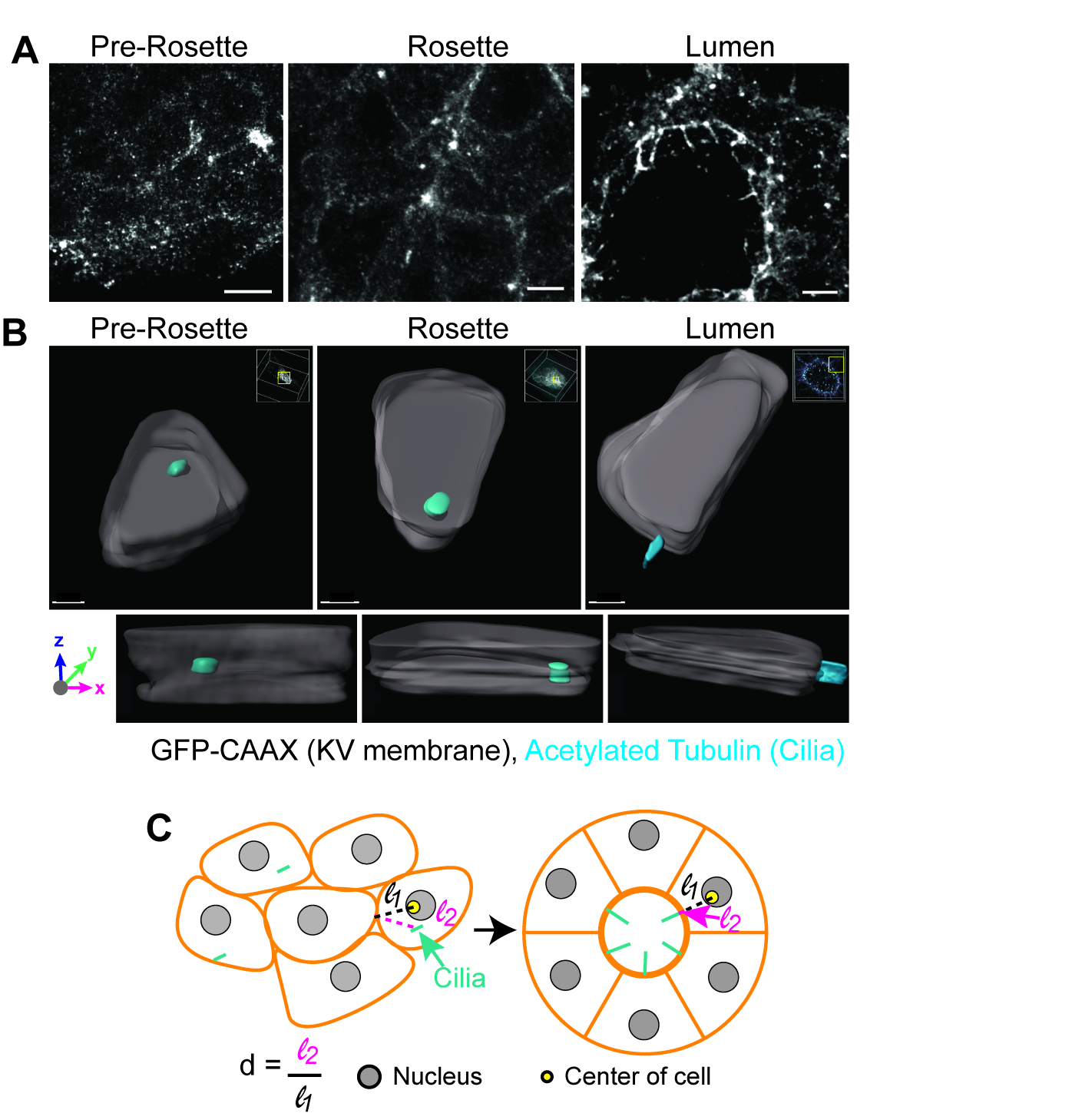

Supplement: S1 Fig — (A) Confocal micrographs showing actin (gray) representatives in Fig 1B. Scale bar, 10 μm. (B) 3D surface rendering of a representative KV cell from pre-rosette, rosette, and lumen KV developmental stages with cilia (acetylated-tubulin, cyan) and KV plasma membranes (KV membranes, Sox17:GFP-CAAX, gray). Refer to S1 Video. Scale bar, 5 μm. (C) Model depicting quantification of relative distance of the cilium from the cell border closest to KV center. Cilia, cyan. Nucleus, gray. Center of KV cells, yellow. Pink dashed line is distance of cilium from cell membrane. Black dashed line is distance of cell center to cell membrane. (TIF) [file pgen.1010765.s004.tif]

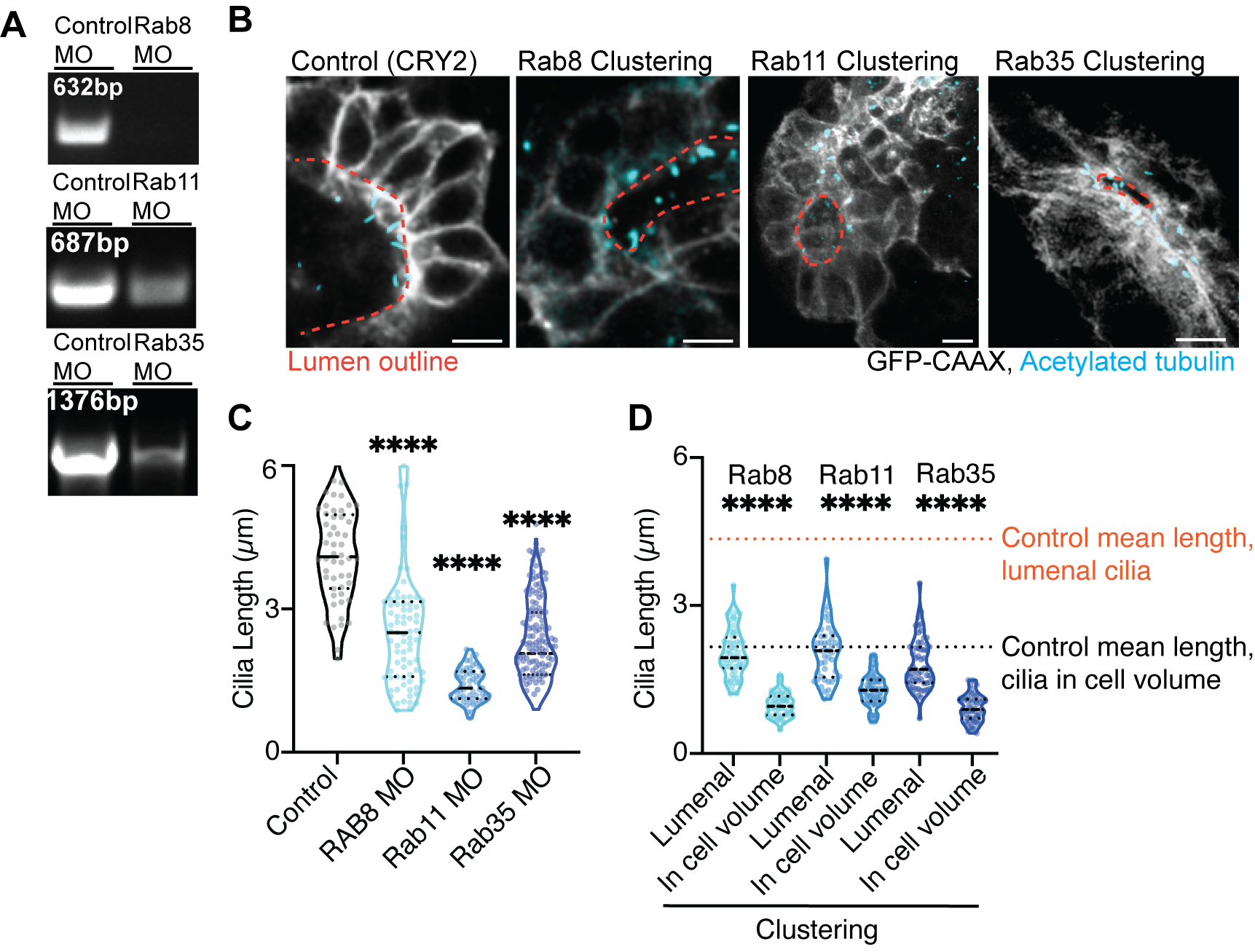

Supplement: S2 Fig — (A) Agarose gel demonstrating RT-PCR of Rab8, Rab11, and Rab35 MO treatment compared to control MO conditions. Amplification of Rab8, Rab11, and Rab35 transcripts shown. NC, negative control. (B) Confocal micrographs of cilia (acetylated tubulin, cyan) in CRY2 (control), Rab8-, Rab11-, and Rab35-clustered Sox17:GFP-CAAX embryos (gray). Clusters not shown. Lumen outline is orange dashed lines. Scale bar, 10 μm. (C) Violin plot depicting cilia length from control (vivo standard control morpholinos from Gene tools), Rab8, Rab11, and Rab35 MO treatment. Dots represent individual cilia length values. Median denoted by line. One-way ANOVA with Dunnett’s multiple comparison test, compared to CRY2. ****p<0.0001. (D) Violin plot depicting length of lumenal cilia compared to cilia in cell volume from Rab8, Rab11, and Rab35 clustering conditions. Dots represent individual cilia length values. Median denoted by line. Dashed lines represent measurements from control embryos denoting average length of lumenal cilia (orange) and average length of cilia in cell volume (black). Unpaired student t-tests. ****p<0.0001. Statistical results detailed in S1 Table. (TIF) [file pgen.1010765.s005.tif]

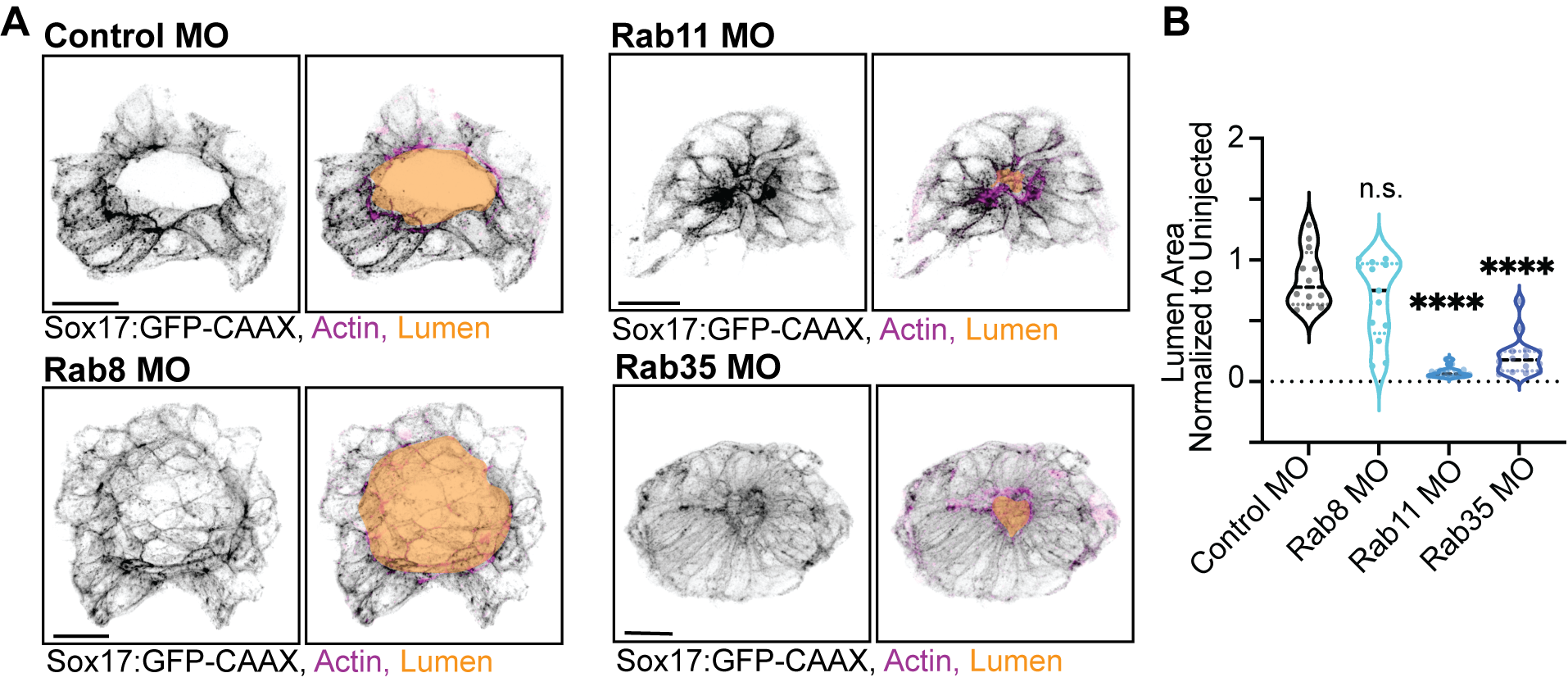

Supplement: S3 Fig — (A) Representative 3D rendering of KV under Rab8, Rab11, and Rab35 MO treatment. Lumen trace (orange), cell membrane (GFP-CAAX, inverted gray), and actin (magenta) shown. Scale bar, 25 μm. (B) Violin plot depicting lumen area normalized to uninjected control values in control (vivo standard control morpholinos from Gene tools), Rab8, Rab11 and Rab35 MO injected embryos. Dots represent individual KV values. Median denoted by line. One-way ANOVA with Dunnett’s multiple comparison test, compared to control MO. n>12 embryos, n.s. not significant, ****p<0.0001. (TIF) [file pgen.1010765.s006.tif]

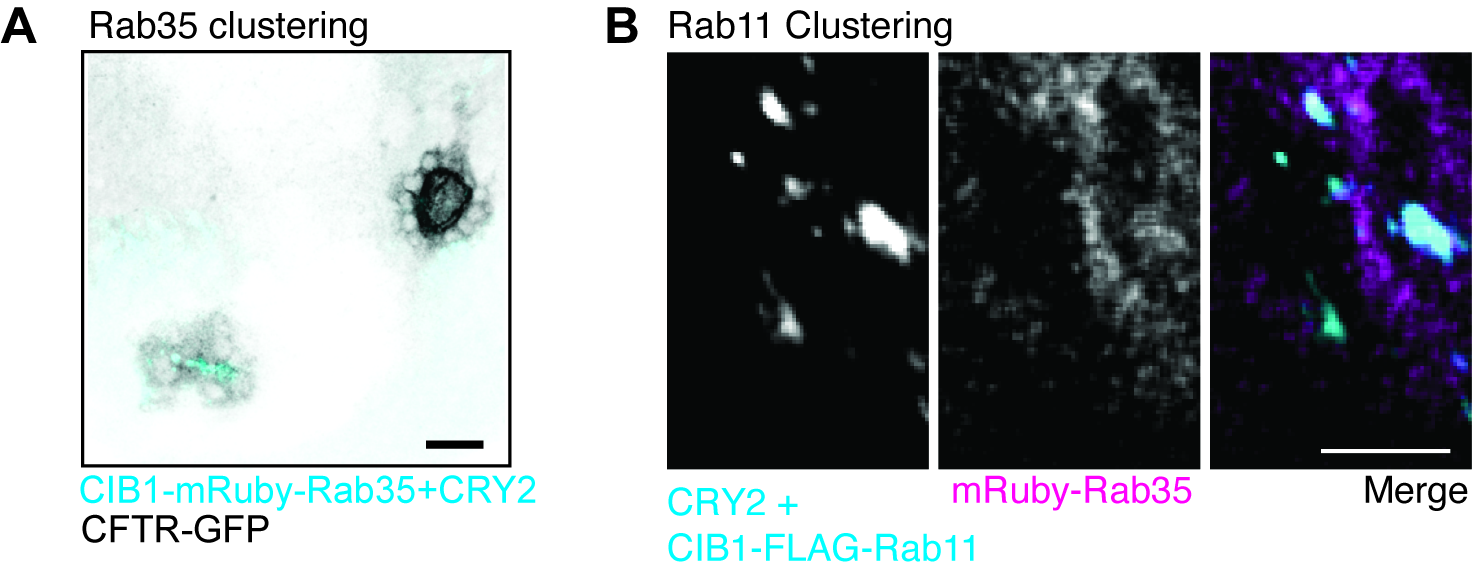

Supplement: S4 Fig — (A) Representative image of optogenetic clustering of Rab35 (cyan) in KV cells; CFTR-GFP (inverted gray) shown. Scale bar, 25 μm. (B) Optogenetic clustering of Rab11 in KV cells. Rab11 clusters (cyan) localization with mRuby-Rab35 (magenta) shown. Scale bar, 7 μm. (TIF) [file pgen.1010765.s007.tif]
