# Supplementary material for: Rab8, Rab11, and Rab35 coordinate lumen and cilia formation during zebrafish left-right organizer development
Source: PLoS Genet. 2023 May 15;19(5):e1010765. doi: 10.1371/journal.pgen.1010765 (PMC10212091; doi:10.1371/journal.pgen.1010765)
Supplement: S1 Table — Figures included are Figs 1D–1G, 4C–4F, S2C–S2D, 5B–5D, S3B, 6B and 6D. (DOCX) [file pgen.1010765.s008.docx]

**Table S1. Detailed statistical analysis of results reported in this study.**

| **Figure** | **Category** | **n**  **cell** | **n**  **embryo** | **n**  **Clutch** | **Statistical Test** | **Parameters** | **Result** | **p-value** |
| --- | --- | --- | --- | --- | --- | --- | --- | --- |
| 1D | Pre-Rosette | N/A | n=7 | n>3 | One Way ANOVA | F (2,31) = 6.917 | ** | 0.0033 |
|  | Rosette | N/A | n=7 |  |  |  |  |  |
|  | Lumen | N/A | n=20 |  |  |  |  |  |
| 1E | KV cells with lumenal cilia across lumen area | N/A | n=29 | n>3 | N/A | N/A | N/A | N/A |
| 1F | N/A | N/A | n=29 | n>3 | N/A | N/A | N/A | N/A |
| 1G | Pre-Rosette | n=8 | n=1 | N/A | One Way ANOVA | F (2,21) = 13.62 | N/A | N/A |
|  | Rosette | n=8 | n=1 |  |  |  | *** | 0.0006 |
|  | Lumen | n=8 | n=1 |  |  |  | *** | 0.0003 |
| 4C | CRY2 | N/A | n=16 | n>2 | One Way ANOVA | F (3, 31) = 12.27 | N/A | N/A |
|  | Rab8 clustering | N/A | n=16 |  |  |  | n.s. | 0.2732 |
|  | Rab11 clustering | N/A | n=5 |  |  |  | **** | <0.0001 |
|  | Rab35 clustering | N/A | n=15 |  |  |  | ** | 0.0009 |
| 4D | CRY2 | N/A | n=16 | n>2 | One Way ANOVA | F (3, 31) = 13.27 | N/A | N/A |
|  | Rab8 clustering | N/A | n=5 |  |  |  | n.s. | 0.8825 |
|  | Rab11 clustering | N/A | n=6 |  |  |  | ** | 0.0070 |
|  | Rab35 clustering | N/A | n=5 |  |  |  | **** | <0.0001 |
| 4E | CRY2 | n=433 | n=17 | n>3 | One Way ANOVA | F (3, 874) = 189.2 | N/A | N/A |
|  | Rab8 clustering | n=145 | n=4 |  |  |  | **** | <0.0001 |
|  | Rab11 clustering | n=61 | n=5 |  |  |  | **** | <0.0001 |
|  | Rab35 clustering | n=239 | n=8 |  |  |  | **** | <0.0001 |
| 4F | CRY2 | n=91 | n=6 | n=3 | One Way ANOVA | F (3, 299) = 46.89 | N/A | N/A |
|  | Rab8 clustering | n=69 | n=6 |  |  |  | n.s. | 0.9545 |
|  | Rab11 clustering | n=49 | n=4 |  |  |  | **** | <0.0001 |
|  | Rab35 clustering | n=94 | n=9 |  |  |  | **** | <0.0001 |
| S2C | Control MO | n=52 | n=3 | n>2 | One Way ANOVA | F (3, 319) = 74.45 | N/A | N/A |
|  | Rab8 MO | n=105 | n=7 |  |  |  | **** | <0.0001 |
|  | Rab11 MO | n=55 | n=3 |  |  |  | **** | <0.0001 |
|  | Rab35 MO | n=111 | n=5 |  |  |  | **** | <0.0001 |
| S2D | Rab8-Lumenal | n=60 | n=3 | n=1 | Unpaired t-test | t=14.98, df=118 | N/A | <0.0001 |
|  | Rab8-In cell volume | n=60 | n=3 |  |  |  | **** |  |
|  | Rab11-Lumenal | n=60 | n=3 |  | Unpaired t-test | t=9.221, df=115 | N/A | <0.0001 |
|  | Rab11-In cell volume | n=46 | n=3 |  |  |  | **** |  |
|  | Rab35-Lumenal | n=71 | n=3 |  | Unpaired t-test | t=11.89, df=118 | N/A | <0.0001 |
|  | Rab35-In cell volume | n=60 | n=3 |  |  |  | **** |  |
| 5B | CRY2 | N/A | n=3 | n=3 | N/A | N/A | N/A | N/A |
|  | Rab8 clustering | N/A | n=3 |  | N/A | N/A | N/A | N/A |
|  | Rab11 clustering | N/A | n=3 |  | N/A | N/A | N/A | N/A |
|  | Rab35 clustering | N/A | n=3 |  | N/A | N/A | N/A | N/A |
| 5C | CRY2 | N/A | n=80 | n>9 | N/A | N/A | N/A | N/A |
|  | Rab8 clustering | N/A | n=72 |  | N/A | N/A | N/A | N/A |
|  | Rab11 clustering | N/A | n=93 |  | N/A | N/A | N/A | N/A |
|  | Rab35 clustering | N/A | n=47 |  | N/A | N/A | N/A | N/A |
| 5D | CRY2 | N/A | n=50 | n=11 | One Way ANOVA | F (3, 94) = 18.43 | N/A | N/A |
|  | Rab8 clustering | N/A | n=9 | n=3 |  |  | **** | <0.0001 |
|  | Rab11 clustering | N/A | n=16 | n=4 |  |  | n.s. | 0.9962 |
|  | Rab35 clustering | N/A | n=23 | n=2 |  |  | **** | <0.0001 |
| S3B | Control MO | N/A | n=12 | n=3 | One Way ANOVA | F (3, 58) = 51.52 | N/A | N/A |
|  | Rab8 MO | N/A | n=13 | n=2 |  |  | n.s. | 0.0839 |
|  | Rab11 MO | N/A | n=21 | n=2 |  |  | **** | <0.0001 |
|  | Rab35 MO | N/A | n=16 | n=4 |  |  | **** | <0.0001 |
| 6B | Rab8 Clustering | N/A | n=8 | n=3 | One Way ANOVA | F (2, 27) = 43.87 | N/A | N/A |
|  | Rab11 Clustering | N/A | n=9 |  |  |  | **** | <0.0001 |
|  | Rab35 Clustering | N/A | n=13 |  |  |  | ** | 0.0015 |
| 6D | Rab11 Clustering-Rab8 | N/A | n=13 | n>2 | One Way ANOVA | F (2, 30) = 63.95 | N/A | N/A |
|  | Rab11 Clustering-Rab35 | N/A | n=9 |  |  |  | **** | <0.0001 |
|  | Rab35  Clustering-Rab11 | N/A | n=13 |  |  |  | **** | <0.0001 |
