# Supplementary material for: Rab8, Rab11, and Rab35 coordinate lumen and cilia formation during zebrafish left-right organizer development
Source: PLoS Genet. 2023 May 15;19(5):e1010765. doi: 10.1371/journal.pgen.1010765 (PMC10212091; doi:10.1371/journal.pgen.1010765)
Supplement: Attachment — Submitted filename: Response_to_Reviewers_HH_AAA.docx [file pgen.1010765.s011.docx]

We thank the reviewers for their comments. We have commented to both Reviewer 1 and 2 below in blue font. All changes made in the manuscript are also highlighted with blue font and denoted within the rebuttal. We argue that the suggested additions and removal of our heart phenotype data (Reviewer 2) have significantly strengthened our manuscript and we thank the Reviewers for their suggestions and critiques.

**Reviewer #1:**

**Summary:** The manuscript by Aljiboury and co-authors examines lumen and cilia formation in Kupffer’s Vesicle (KV), the zebrafish laterality organ, with a specific focus on the roles of the small GTPases Rab8, 11 and 35. In mammalian tissue culture systems all 3 Rabs have been implicated in ciliogenesis and/or lumen formation. In this work, the authors show that Rab35 and Rab11 play key roles in lumen formation while Rab8 is not required. They show that Rab8 is important for cilia elongation. The authors also provide convincing evidence that cilia in the KV are formed inside the cell via a pathway they term the intracellular pathway. The authors use a combination of experimental approaches to produce high quality data and the findings should be of broad interest to cell and developmental biologists. Some minor suggestions are outlined below.

**Suggestions:**

1. page 3 line 20. The authors refer to the source of KV cells as being the endoderm. The KV comes from a subset of dorsal EVL cells (Oteiza et al., 2008; Warga and Kane, 2018). While the DFCs express both endodermal and mesodermal markers, after KV breakdown cells contribute to mesodermal structures (notochord and somites) (Melby et al., 1996). The common view in the field is that the germ layer identity of DFCs is ambiguous (Stainier and Warga 2002), so I suggest removing or qualifying statement the that the KV comes from the endoderm.

We thank Reviewer 1 for their comment. We have edited the main text in the introduction to clarify these points. Refer to the main text for edits.

2. The legends for Figures 1 and 2 are rather terse—it would be helpful to add some detail so that the reader does not have to refer to the main text to glean the major points.

We have reshaped the figures and legends in response to this comment and Reviewer 2’s comments. We have also included more details in the new figure legends. We argue that the reshaped figures and figure legends are now easier to read and main conclusions easily attained.

3. In Fig S1D the actin in black is not visible.

To address this comment, we have added the actin channel in Figure S1A for the three KV stages so that it is easier to visualize cell boundaries marked by actin highlighted in Figure 1B. We refer to this in the main text.

**Reviewer #2:**

**Summary:** This manuscript has investigated the roles of three Rabs in controlling the formation of a ciliated Kupffer's vesicle, the transient structure that forms in the zebrafish tailbud where left-right asymmetry is thought to be first established. The authors use a range of immunostaining and live imaging, together with optogenetic and morpholino loss-of-function studies to demonstrate roles for Rabs in lumen expansion and proper cilia formation. They then demonstrate that the lumen expansion defect is likely due to failure of trafficking CFTR to the apical membrane. In the last section of the manuscript, they perform some left-right patterning phenotyping and find defects when Rabs are disrupted. This manuscript contains high quality data and makes some novel findings. As such, I am overall supportive of publication. However, there were two major concerns: 1) the manuscript was very difficult to read and; 2) I am not convinced the left-right heart phenotypes reflect issues at KV. I detail these concerns, plus some other more minor issues, below:

**Major comments:**

1. This was a challenging paper to read, as the writing is dense, the narrative jumps around, some critical background information is not provided and the main body of the text is not broken into defined results. For instance, the first paragraph of the results is about Rab localization but then the manuscript departs from that topic to discuss the serendipitous observation of intracellular ciliogenesis. It is not until much later that we return to Rabs and perform functional experiments.
   Recommendations:

1) Reorder some of the results to walk the reader more logically through the data

2) break the results section into subheadings to help the reader get the main point of what is described in the text in that section

3) Add some background information on Rab GTPases and their potential roles in the Introduction. Many readers will not be automatically familiar with Rab pathways.

We thank the reviewer for their suggestions and have incorporated them in our resubmission. We have reordered the main text and figures so that it flows more logically. Additionally, we have broken up the results into subsections leading with subheadings to conclude the main findings that parallel to the conclusions in the figures. Lastly, we have included a more thorough discussion and background information of what is previously known about the roles of Rab8, Rab11 and Rab35. We believe that these changes have significantly improved the flow and readability of the paper, which will make it easier for the readers to grasp the main conclusions and follow the logic of the experiments performed.

2. Rab localization studies use overexpression of tagged mRNAs. How do we know the localization we are seeing is not caused by overexpression?

In the case of Rab11 we have an endogenously expressed GFP-tagged Rab11 line that shows similar localization to ectopically expressed GFP-Rab11, see updated Figure 2C-D for direct comparison and clarification in text. These representative images suggests that ectopic expression of the Rab11 GTPase does not affect its localization in the KV. While we do not currently have endogenously tagged Rab8 or Rab35 zebrafish transgenic lines, we argue that Rab8 and Rab35 localization will be similarly unaffected by ectopic expression. In addition, we have attempted to immunostain these GTPases in the zebrafish embryo to address Reviewer 1’s concern but have had little success in doing so due to most of our GTPase antibodies targeting the human protein. Thus, this remains to be a concern. Our goal is to obtain endogenously tagged versions of all these GTPases for our future studies.

3. The interpretation of the left-right patterning phenotype in Figure 4 is not very convincing given how developmentally abnormal some of the embryos are more broadly. How can the authors say the abnormal heart looping phenotype is caused by defective KV lumen expansion/cilia phenotypes specifically when the embryos are so damaged? Is the LPM abnormal, are the heart cells themselves abnormal? This is especially important since the prevalence of abnormal looping seems quite high (even higher than what you see when Nodal is removed) and higher than what you'd expect based on their more cellular phenotyping. Also note, it has been shown that heart looping is mostly controlled by heart-intrinsic effects and not solely by KV. They are shining blue light on embryos until 12 hpf, well after the stage cardiac precursor cells have formed and are migrating. These cells likely use Rabs for various functions. Recommendation: Either more convincingly show L-R defects (additional asymmetric organs, Nodal/Pitx2 LPM staining), and show that they originate in KV (e.g. target Rab MO's to KV specifically), or perhaps remove this section from the manuscript.

These are important concerns, and we thank the reviewer for noting them. We also agree that this data maybe too preliminary and have taken Reviewer 2’s advice to remove these studies from the manuscript and more clearly lay out our other points. We believe that removing these studies and focusing on the other main conclusions addressed by this manuscript has significantly improved the study.

**Minor comments:**

1. Line 3-4 pg 4 "…beat in a leftward motion…" This is an imprecise. Cilia generate a directional flow but they do not 'beat leftward'. Recommendation: re-phrase.

We have edited the text to clarify that KV cilia beating generates a directional fluid flow required for symmetry breaking of the animal and establishing left-right asymmetry. See main text for more details.

2. There are many typos in the manuscript e.g. in the abstract, line 6 "to establishment" and pg 4 line 15 "…similar extent that it does…". There are many others, making for a distracted read. Recommendation: proof read and correct.

We thank the reviewer for their comment. We have proof-read and corrected the typos in the manuscript.

3. At the end of page 7 and start of page 8, the writing is confusing. Are all cilia shorter in optogenetic clustered conditions, or only those "stuck in the cell volume"? (This is explained for Rab8, but not the others). Recommendation: re-phrase/explain more thoroughly.

All cilia on average are shorter, the cilia that are stuck in the cell volume under clustering conditions are significantly shorter than the ones that can extend into the lumen (please refer to new Figure panel S2D). In addition, the cilia that remain in the cell volume under clustering conditions are also significantly shorter to control KV cilia that are in the cell volume during early KV development (Figure S2D). We argue that cilia can only extend to a certain length when inside the cell, and then reach its final length when it is extending into the lumen. We think that Rab11, Rab8, and Rab35 are all involved in general building of the cilium. We have edited the main text to explain this more clearly.

4. Are the cilia length defects a knock-on consequence of failure to expand the lumen? Or do Rab35 and Rab11 play specific roles in elongating cilia in addition to expanding the lumen? Recommendation: test/mention whether CFTR mutation causes the cilia length/extension phenotype in addition to the lumen expansion phenotype, or whether these phenotypes are separable under CFTR loss-of-function conditions.

We thank the reviewer for this response, and in response to this we explored whether the defects found with CFTR and lumen expansion relate to cilia formation. Interestingly, detailed studies performed in Jeffrey Amack’s lab demonstrated that CFTR mutations (*pd1048*) and treatment with morpholinos against CFTR caused no defects in cilia formation but did result in lumen area defects at the 8SS (Gokey JJ *et al.* Developmental Dynamics 2016). On average lumen areas were approximately 1000 μm^2^ in *cftr^pd1048^* mutant embryos compared to 3000μm^2^ in control embryos. What we argue is that CFTR defects result in inhibition of lumen expansion, however this does not directly affect cilia formation. In addition, we do not find lumen expansion defects with Rab8 clustering or depletion (MO) conditions. However, we do find cilia length defects under the same Rab8 disruption methods that further suggests cilia length is not completely dependent on lumen area dynamics. We think that Rab11 and Rab35 may have an integral role in lumen formation due to the increased incidence of embryos stuck in pre-rosette or rosette stages with Rab11 and Rab35 clustering (Figure 5C). We also argue that they have significant roles in building of a cilium, since a significant proportion of cells are unable to assemble a cilium (Figure 4C). If, and when, embryos do form a KV with a lumen, then we observe severe defects in CFTR trafficking (Figure 6A-B) inhibiting the ability of the KV to expand its lumen. We have included a more thorough discussion of this in the discussion.

One thing we are thinking about now is if these early trafficking events to the mother centriole, potentially involving Rab11 and Rab35, to build a cilium are required for lumen formation or is it that Rab11 and Rab35 have two independent roles one in cilia formation and one in lumen formation. These are studies we hope to follow up on in the future and have now included in our discussion.

5. Is the curved tail phenotype always upward curl? Motile cilia are essential for axial straightening, but usually motile cilia mutants have downward curled tails. Do the authors think the origin of the tail curl is cilia phenotypes or something else? Recommendation: describe more precisely the direction of tail curl phenotypes.

Due to Figure 4 being too preliminary and Reviewer 2’s recommendation to remove these studies, this observation has been removed. Our future work on this will take tail curve directionality into account based on Reviewer 2’s comments.
